# A Disintegrin and Metalloprotease 15 (ADAM15) as a Potential Predictor of Distant Metastasis in Colorectal Cancer (CRC)

**DOI:** 10.3390/jcm14145082

**Published:** 2025-07-17

**Authors:** Adrianna Romanowicz, Marta Łukaszewicz-Zając, Barbara Choromańska, Sara Pączek, Hady Razak Hady, Piotr Myśliwiec, Jacek Jamiołkowski, Piotr Stępniewski, Leszek Kozłowski, Barbara Mroczko

**Affiliations:** 1 Department of Biochemical Diagnostics, Medical University, 15-269 Białystok, Polandmroczko@umb.edu.pl (B.M.); 2 Department of Neurodegeneration Diagnostics, Medical University of Bialystok, 15-269 Białystok, Poland; 3 First Department of General Surgery, Medical University, 15-267 Białystok, Poland; 4 Second Department of General Surgery, Medical University, 15-267 Bialystok, Poland; 5 Population Research Center, Medical University, 15-089 Bialystok, Poland; 6 Oncology Center of Bialystok, 15-027 Bialystok, Poland

**Keywords:** a disintegrin and metalloprotease, biomarker, colorectal cancer

## Abstract

**Background:** The pro-tumorigenic role of a disintegrin and metalloprotease 15 (ADAM15) is supported by its modified expression in primary tumors and ability to promote tumor growth in colorectal cancer (CRC). Cancer cell-derived ADAM15 promotes the progression of this malignancy by modulating the tumor microenvironment. However, according to our knowledge, this study is the first to assess serum ADAM15 concentrations in CRC patients in comparison to classical tumor markers—carcinoembryonic antigen (CEA) and cancer antigen 19-9 (CA19-9)—and a marker of the inflammatory process, C-reactive protein (CRP). The aim was to evaluate whether circulating serum ADAM15 might be a candidate biomarker for CRC diagnosis and progression. **Methods:** The study included 110 CRC patients and 54 healthy volunteers. Serum concentrations of ADAM15, CEA, and CA19-9 were measured using immunoenzyme assays, while CRP levels were assessed by the turbidimetric method. Diagnostic characteristics of all tested proteins were calculated. **Results:** Serum ADAM15 and classical tumor marker (CEA and CA19) levels were higher in CRC patients than in healthy subjects. However, a significant difference was observed only for CEA (*p* < 0.001). ADAM15 concentrations were significantly higher in CRC patients with distant metastases compared to those without metastases (*p* = 0.043). The highest diagnostic sensitivity (89%) was achieved by combined analysis of ADAM15 and CRP levels. **Conclusions:** These findings suggest a significant role of ADAM15 in CRC pathogenesis, indicating the usefulness of this protein in the prediction of distant metastases. Measurement of serum ADAM15, especially in combination with classical tumor markers (CEA) and inflammation markers (CRP), may improve the diagnosis of patients with CRC.

## 1. Introduction

The incidence of colorectal cancer (CRC) will continue to increase during the next two decades due to continuous exposure to risk factors such as unfavorable diet, obesity, alcohol, smoking, and low physical activity as well as the expected ageing of the population [1]. The mortality and morbidity of CRC are ranked fourth and third among malignant tumors, respectively [2]. Knowledge of trends in incidence of this malignancy and the clinical characteristics of CRC patients is imperative to diagnosis and treatment, as well as in the development of a strategy to assess future changes in the patient population. The focus on the primary prevention of CRC including screening programs and early diagnosis of patients such as biochemical markers will reduce CRC’s incidence and decrease the mortality rate of patients with this common malignancy. Unfortunately, CRC is detected in advanced stages of tumor development, mostly based on the presence of the symptoms that appear [3]. Therefore, new biomarkers are sorely needed. Carcinoembryonic antigen (CEA) and carbohydrate antigen 19-9 (CA 19-9) have been established as the most validated serum markers; however, their usefulness in the detection of CRC is unsatisfactory due to their low diagnostic sensitivity and specificity in the early stages.

Tumor progression is highly affected by interactions between tumor cells and the tumor microenvironment (TME). It has been proven that key molecules involved in the communication of cancer cells with the TME are released to the extracellular space by secretion or cleavage from the cell surface [4,5,6,7]. ADAM15 is a type I transmembrane protein belonging to the a disintegrin and metalloprotease (ADAM) family, characterized by the presence of metalloproteinase, disintegrin, cysteine-rich, and cytoplasmic domains. It is primarily expressed on the cell membrane, but its extracellular domain can undergo proteolytic cleavage and be released into the extracellular space or bloodstream—a process known as ectodomain shedding. This soluble form of ADAM15 can be detected in serum and may reflect pathological processes within the tumor microenvironment, including tumor–host interactions. Ectodomain shedding is regulated by various factors such as protease activity, inflammation, and cellular stress. In the context of tumor metastasis, shedding may be enhanced due to increased matrix remodeling and interactions with stromal components. Therefore, elevated levels of soluble ADAM15 in the serum may not only indicate tumor presence but also dynamic changes related to metastatic progression [7]. The pro-tumorigenic role of ADAMs has been confirmed in murine cancer models as well as in vitro, supporting their promoting role in tumor growth and metastasis.

Among ADAMs that exhibit a functional protease domain, ADAM15 is proved to be upregulated in the most common cancer types, including CRC [4,8,9,10]. Some clinical investigations have reported that this molecule is involved in cell–ECM and cell–cell communication, and thus may promote proliferation and invasion in vitro as well as support tumor growth and metastasis in murine cancer models [10,11,12,13,14]. It has been shown that 63% of CRC cells demonstrate reduced expression of ADAM15 in cancer cells, which has been evaluated at the mRNA level. Moreover, the downregulation of this molecule is associated with histologically poorly differentiated malignancies. Some investigators assessed ADAM15 expression in colon carcinomas using both IHC and mRNA quantitative methods [15,16]. The authors observed decreased expression of ADAM15 in CRC associated with a loss of differentiation in a subset of colon carcinomas, which indicates that the role of ADAM15 in cancer progression is tissue-specific [15]. Despite the fact that the implication of ADAMs in cell–ECM and cell–cell interactions has been reported in vitro using the evaluation of tissue expression of this proteases in CRC cells [17,18], the concentrations of ADAM15 in the sera of CRC patients and its potential diagnostic significance is scarcely explored.

However, to the best of our knowledge, the present study is the first to demonstrate serum concentrations of ADAM15 in relation to well-established tumor markers for CRC, such as CEA and Ca 19-9, as well as a marker of inflammation—C-reactive protein (CRP). Therefore, the goal of our study was to assess the usefulness of serum ADAM15 as a potential novel tumor marker of CRC in comparison to well-established tumor markers such as CEA and CA 19-9, as well as CRP. Moreover, the association between the serum levels of the tested proteins and the clinicopathological characteristics of the tumor were assessed, such as the tumor stage according to the TNM classification, the depth of tumor invasion (T factor), and the presence of lymph nodes (N factor) and distant metastases (M factor). In addition, the diagnostic usefulness was also calculated based on diagnostic sensitivity and specificity, accuracy, and predictive values for negative (NPV) and positive (PPV) results, as well as the areas under the ROC curve (AUC), for ADAM15 in comparison to the other tested proteins (CEA, CA19-9, and CRP). The presented investigation is the continuation of our previous studies evaluating the serum levels of other proteases such as matrix metalloproteinases and their tissue inhibitors as potential tumor markers for CRC [19,20].

## 2. Patients and Methods

The total study group consisted of 164 patients, including 110 patients with colorectal cancer (28 women and 82 men) and a control group of 54 healthy volunteers (31 women and 23 men). The patients were diagnosed in the First Department of General Surgery, Medical University of Bialystok in Poland, or the Bialystok Oncology Centre (Poland). The clinical diagnosis of CRC was based on the microscopic examination of tissue samples. Colorectal cancer was staged based on the TNM (tumor–node–metastasis) classification, presented by the International Union Against Cancer (UICC). All patients with CRC were divided into four subgroups, dependent on the tumor stage (TNM), depth of tumor invasion (T factor), presence of lymph node metastasis (N factor) and distant metastasis (M factor), and tumor differentiation (G0, G1, G2, and G3). The characteristics of the CRC patients are presented in Table 1.

The study was conducted in accordance with the Declaration of Helsinki. Informed consent was obtained from all the patients and the present project was approved by the Local Bioethics Committee (R-I-002/284/2017) of the Medical University of Bialystok. All samples were anonymized.

The following exclusion criteria were applied: no active infections and a lack of symptoms of an infection, the absence of comorbidities that could affect protein concentrations (respiratory diseases and digestive tract diseases), and the absence of systemic diseases such as lupus and rheumatoid arthritis. All study participants had a BMI within the healthy weight range in order to exclude obesity-related inflammatory factors.

Blood samples were collected from patients between August 2019 and March 2020 (Sarstedt, Nümbrecht, Germany) prior to treatment and stored at −80 °C until analysis (June 2021, March 2022). Serum ADAM15 levels were measured using enzyme-linked immunosorbent assay kits (ELISA) (Innovative Research, Inc, Novi, MI, USA) according to the manufacturers’ instructions. Serum concentrations of classical tumor markers such cancer antigen 19-9 (CA 19-9) and carcinoembryonic antigen (CEA) were examined by the chemiluminescent microparticle immunoassay (CMIA) method (Abbott, 675 N. Field Drive Lake Forest, IL 60045, USA) using the ARCHITECT 8200 ci (Abbott, Chicago, IL, USA), while serum CRP levels were measured by the immunoturbidimetric method (Abbott, 675 N. Field Drive Lake Forest, IL 60045, USA) using the ARCHITECT 8200 ci. Youden’s index was used to select optimal predicted probability cut-off values. The reference cut-off values were as follows: 972,246 pg/mL for ADAM15; 2.32 ng/mL for the classical tumor marker CEA; 17.59 U/mL for the classical tumor marker CA 19-9; and 3.75 mg/l for CRP.

### Statistical Analysis

Serum ADAM15, CA 19-9, CEA, and CRP concentrations did not follow a normal distribution in the preliminary statistical analysis (χ^2^-test); therefore, nonparametric statistical methods were applied. The Mann–Whitney U test was utilized to compare two groups, while the Kruskal–Wallis test was used for comparisons among three or more groups. In addition, the post hoc Dwass–Steele–Critchlow–Fligner test was employed if significant differences were found. Moreover, to assess the diagnostic significance of the proteins tested, diagnostic criteria were calculated such as diagnostic sensitivity and negative (NPV) results, and the AUC values were calculated. Differences were considered statistically significant when *p* < 0.05. Microsoft Office Excel 2019 MSO was employed to calculate diagnostic parameters and IBM SPSS Statistics 20.0 was used for statistical analysis. Furthermore, logistic regression was used to assess the strength of the association between various risk factors and CRC. In the first step of the analysis, univariate logistic regression models were used to indicate the relationship between each variable and CRC risk. Secondly, variables where *p* < 0.05 were included into the multivariate model. Finally, the least significant variables were removed from the model in a stepwise manner based on the Wald statistic.

## 3. Results

Serum concentrations of ADAM15 and classical tumor markers (CEA and CA 19-9), as well as the marker of inflammation CRP, were higher in CRC patients in comparison to healthy controls; however, these differences were significant only for serum CEA (*p* < 0.001) and CRP (*p* < 0.001) levels (Table 2).

Following analysis of the association between the serum levels of the analyzed proteins and the tumor stage of CRC according to the TNM classification, the concentrations of ADAM15, as well as the classical tumor markers CEA and CRP, were the highest in stage IV CRC, but the difference was significant only for CEA (*p* = 0.049) and CRP (*p* = 0.013). In addition, CRP concentrations were significantly lower in CRC patients in stage I (*p* = 0.024) and stage II (*p* = 0.048) in comparison to CRC subjects in stage IV, which was calculated using a post hoc wg Dwass–Steele–Critchlow–Fligner test (Table 3).

Taking into consideration the relationship between the serum levels of the analyzed proteins and the clinicopathological features of CRC, we reported that ADAM15, CEA, and CRP levels were elevated in subjects with deeper tumor invasion (T3 and T4 subgroups) when compared with individuals from the T1 + 2 subgroup (Table 4). Moreover, ADAM15, CEA, CA 19-9, and CRP levels were higher in the sera of patients with the presence of lymph node metastasis (N1 subgroup) than in subjects without nodal involvement (N0 subjects); however, statistically significant differences were found only for CEA (*p* = 0.012) and CRP (*p* = 0.006) levels (Table 4). In addition, we revealed that serum ADAM15 concentrations were significantly higher in CRC patients with the presence of distant metastasis (M1 subgroup) in comparison to the M0 subgroup (*p* = 0.043), similar to the CEA (*p* = 0.21) and CRP (*p* = 0.004) levels (Table 4). The statistical differences between different stratified groups (TNM stage, T, N, and M factor) and the healthy controls are presented in Table 5.

**Figure 1:**
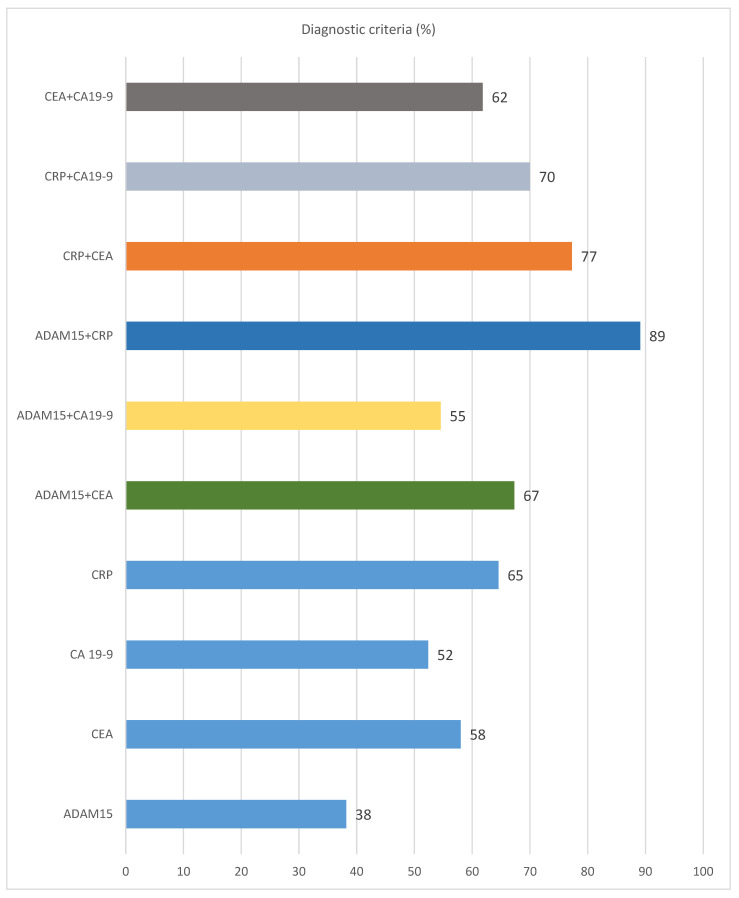
The percentage of elevated concentrations of ADAM15 and well-established tumor markers, as well as C-reactive protein levels in colorectal cancer patients.

Diagnostic criteria such as the diagnostic sensitivity and specificity, accuracy, and predictive values for negative (NPV) and positive (PPV) results, as well as the areas under the ROC curve (AUC), were calculated to evaluate the diagnostic usefulness of ADAM15 in CRC patients (Table 5 and Figure 1). Moreover, the diagnostic relevance of this protein was compared with well-established tumor markers for CRC including CA19-9 and CEA, as well as a marker of inflammation—CRP. The diagnostic sensitivity of ADAM15 (38%) was lower than for the classical tumor markers (CA 19-9—52%, CEA—58%) and CRP (65%). The combined analysis of ADAM15 and CEA increased the diagnostic sensitivity up to 67% and was higher than the assessment of classical tumor markers in combination (CEA + CA19-9 = 62%) (Table 6 and Figure 1).

However, the highest percentage of elevated results was reported for the combined analysis of ADAM15 with CRP (89%) (Table 5 and Figure 1). Moreover, it was indicated that some of the tumor patients (26%) who were negative for CRP presented positive concentrations of ADAM15. The diagnostic specificity of the serum ADAM15 level measurement was equal to those of CEA (81%) and lower in comparison to CA19-9 (96%) and CRP (91%). The combination of ADAM15 and CRP yielded reduced diagnostic specificity com-pared to the individual use of each marker. The predictive value of positive results (PPV) of ADAM15 (81%) was comparable (CEA—85%, CA19-9—92%) and CRP (93%). The predictive value of negative results (NPV) for ADAM15 (39%) measurement was higher than for CA19-9 (37%), but lower in comparison to CEA (46%) and CRP (56%). The highest value of NPV was calculated for the combined analysis of ADAM15 and CRP (77%). The diagnostic accuracy (ACC) for ADAM15 (52%) was higher than for CA19-9 (45%), but lower when compared to CEA (62%) and CRP (73%). Similar to NPP, for the value of ACC the highest results were obtained for the combined measurement of ADAM15 and CRP (84%) (Table 5 and Figure 1).

**Figure 2:**
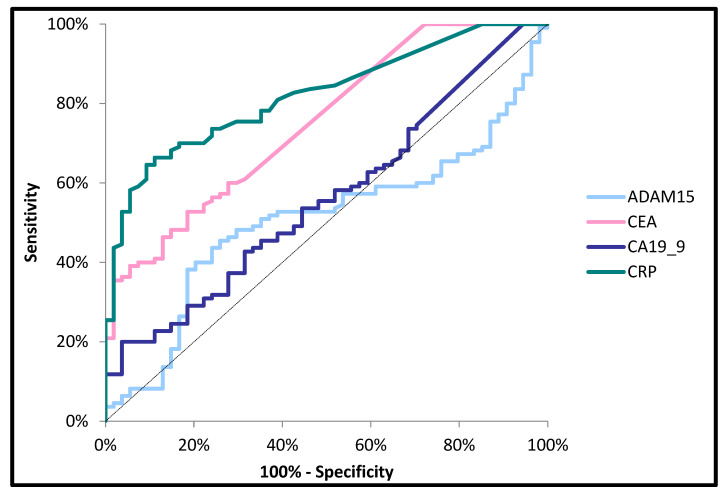
Areas under ROC curves (AUC) for ADAM15 (0.5070. *p* > 0.005), CA 19-9 (0.5617. *p* > 0.005), CEA (0.7519. *p* < 0.001), and CRP (0.8220. *p* < 0.001) in colorectal cancer patients.

**Figure 3:**
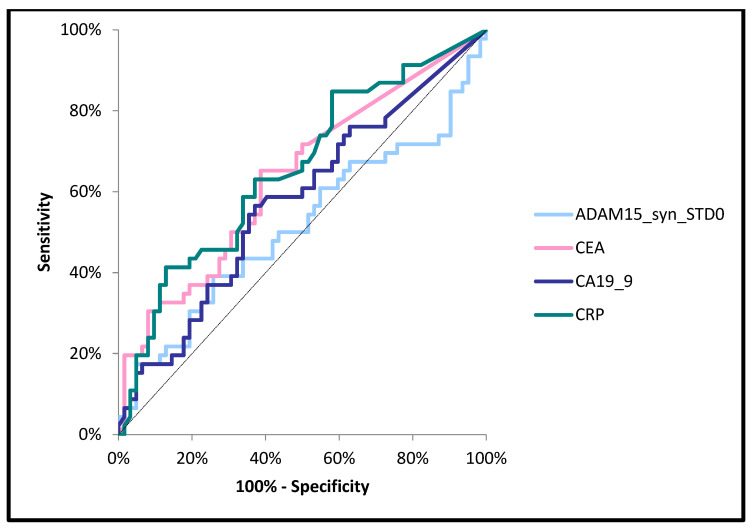
Areas under ROC curves (AUC) for ADAM15 (0.5140, *p* > 0.05), CA 19-9 (0.5768, *p* > 0.05), CEA (0.7519, *p* < 0.001), and CRP (0.6548, *p* = 0.004) in differentiation between patients with nodal involvement and patients without nodal involvement.

**Figure 4:**
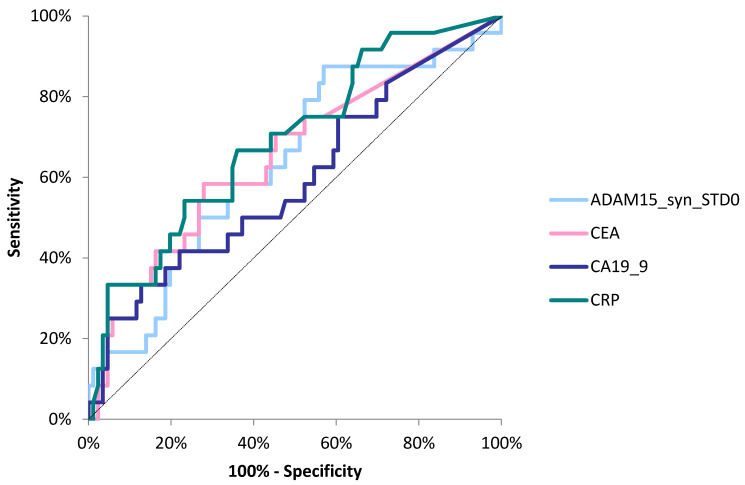
Areas under ROC curves (AUC) for ADAM15 (0.6357, *p* = 0.04), CA 19-9 (0.5928, *p* > 0.05), CEA (0.6373, *p* = 0.01), and CRP (0.6899, *p* = 0.002) in differentiation between subjects with the presence of distant metastases and patients without distant metastases.

The area under the ROC curve (AUC) for ADAM15 (0.5070, *p* > 0.05) was lower than for the classical tumor markers (CA19-9—0.5617, *p* > 0.05; CEA—0.7519, *p* < 0.001) and the marker of inflammation (CRP—0.8220, *p* < 0.001) in the diagnosis of CRC (Figure 2). Moreover, the AUC for ADAM15 (0.5140, *p* > 0.05) was lower than that for CA19-9 (0.5768, *p* > 0.05), CRP (0.6548, *p* = 0.004), and CEA (0.6373, *p* = 0.01) when differentiating between patients with nodal involvement and patients without nodal involvement (N0) (Figure 3). In addition, the AUC for ADAM15 (0.6357, *p* = 0.04) was higher than for CA19-9 (0.5928, *p* > 0.05), and slightly lower in comparison to CEA (0.6497, *p* = 0.023) and CRP (0.6899, *p* = 0.002) in the differentiation between subjects with the presence of distant metastases and patients without distant metastases (Figure 4). The cut-off values of all the tested proteins were estimated using the Youden index.

The relationship between various risk factors and CRC risk was first examined in univariate analysis in order to identify risk factors that qualify for the multivariate model (results were presented as *p* values and odd ratios—ORs). Among all proteins tested, only the concentrations of CEA (*p* = 0.004, OR = 1.428) and CRP (*p* < 0.001, OR = 1.392) were associated with significantly increased CRC prediction, which was also proved in the multivariate model of CEA (*p* = 0.018, OR = 1.352) and CRP (*p* = 0.001, OR = 1.341) (Table 7).

## 4. Discussion

Colorectal cancer remains the fifth leading cause of cancer [21]. The incidence rate of this malignancy has increased in the last few years. Therefore, low-cost, non-invasive methods including the assessment of novel biomarkers useful in early diagnosis are sorely needed. It was proved that tumor progression and response to treatment are highly affected by interactions between cancer cells and the tumor microenvironment (TME). Various signaling receptors and soluble factors involved in this crosstalk might be shed by a family of ADAM proteins. Some investigations reported that the upregulation of ADAM15 has been linked to worse survival in cancer patients and a tumor-promoting function both in vitro and in murine cancer models [2,4]. Moreover, the catalytic activity of ADAM15 promotes the shedding of several key regulatory factors to the extracellular space such as cell signaling factors and molecules involved in cell adhesion and cell–cell communication [2,3,4,22,23,24].

A growing body of evidence has reported the upregulation of ADAM15 expression in the pathogenesis of CRC. However, our study as the first to evaluate the significance of serum ADAM15 as a candidate tumor marker in the diagnosis and progression of CRC. We aimed to assess the association between serum levels of ADAM15 and the clinicopathological characteristics of CRC, as well as its potential significance as a candidate tumor marker in the diagnosis of CRC patients. The current study is a continuation of our previous research, which focused on the role of other proteases such as metalloproteinases (MMP-9 and MMP-2) and their tissue inhibitors (TIMP-1 and TIMP-2) as candidates for tumor markers in gastro-intestinal malignancies, including CRC [19,20].

In our investigation, we reported that serum ADAM15 concentrations were higher in CRC patients compared to healthy controls, similar to the classical tumor markers and CRP; however, significant differences were only observed for CEA and CRP. Although we did not determine the tissue expression of ADAM15, its increase in serum concentration is probably due to tissue changes. Indeed, Puig-Blasco et al. [4] reported that ADAM15 expression is increased in colon and rectal cancer tissues compared to normal tissues. However, the authors assessed ADAM15 expression in colon and rectal cancer tissues using the Gene Expression Profiling Interactive Analysis 2 (GEPIA2 2019) tool and data available in The Cancer Genome Atlas (TCGA) [4,25]. Similar findings were presented by Yamada et al. [26] who also demonstrated significantly higher levels of ADAM15 mRNA in cancer cells in comparison to normal epithelial cells; however, this study was performed in pancreatic cancer patients using the quantitative real-time reverse transcription polymerase chain reaction [26]. Moreover, ADAM15 was highly expressed in hepatocellular carcinoma and lung cancer tissues compared with corresponding noncancerous tissues based on the analysis of RT-qPCR, or Western blot and IHC [27]. The authors confirmed that ADAM15 served a crucial role in the progress of HCC and non-small cell lung cancer [24,27]. Our findings and the results of other authors suggest that CRC cells are able to produce ADAM15.

In our present study, the association between serum ADAM15 levels and the tumor stage of CRC according to the TNM classification, as well as clinicopathological characteristics of CRC, was investigated. The concentrations of ADAM15, as well as the classical tumor markers and CRP, were the highest in the advanced stage of CRC and in patients with the presence of lymph node metastasis (N1 subgroup) than in subjects without nodal involvement (N0 subjects) and an early stage of CRC; however, statistically significant differences were only found for the CEA and CRP levels. In addition, we revealed that serum ADAM15 concentrations were significantly higher in CRC patients with the presence of distant metastasis (M1 subgroup) in comparison to the M0 subgroup, similar to the CEA and CRP levels. ADAM15 is proposed to play a dual role in cancer metastasis, not only by leveraging its disintegrin and metalloproteinase domains but also by promoting tumor cell migration and angiogenesis. Through its disintegrin domain, ADAM15 facilitates the detachment of cells from the extracellular matrix (ECM), while its metalloproteinase domain enables the degradation of ECM components. It has been demonstrated to degrade collagens I and IV and cleave the pro-inflammatory cytokine CD23, further contributing to the remodeling of the tumor microenvironment and facilitating metastatic progression [28,29]. Based on our results, we may conclude that elevated serum ADAM15 levels may serve as a predictor of distant metastasis in CRC. Our findings are similar to those of Lucas et al., who determined ADAM15 levels in prostate cancer patients [30]. The authors confirmed that ADAM15 mRNA and protein levels were elevated in prostate cancer cells, and its expression was found to be significantly higher during metastatic progression [30]. Moreover, ADAM15 cDNA and protein levels were significantly increased in prostate tumor tissue and correlated with metastatic disease progression [31]. These results suggest that ADAM15 may play an important role in the malignant progression of prostate tumors. Contrasting results were presented by Yamada et al., who failed to find any correlation between ADAM15 levels and tumor progression pattern and found no relation between the level of ADAM15 mRNA and the status of lymph node metastasis, prognosis, or histological differentiation in pancreatic cancer [26]. Other authors have analyzed the association of ADAM15 expression with the clinicopathological characteristics of patients with non-small cell lung cancer [24]. The investigators reported that ADAM15 expression was significantly associated with lymph node metastasis; however, no statistical correlations were observed between ADAM15 expression and the patients’ age, gender, histologic types, tumor differentiation stages, or TNM stages [24].

Some clinical investigation reveals a novel mechanism of ADAM15 in promoting cancer cell invasion through directly targeting MMP9. The authors proved that ADAM15 interacts with MMP9 and promotes the maturation and activation of pro-MMP9 through a proteolytic mechanism [24]. In our previous study, we investigated serum levels of MMP-9 in CRC patients [19]. The concentrations of MMP-9, similarly to those of the classical tumor markers (CA 19-9 and CEA), were significantly higher in the sera of CRC patients in comparison to healthy subjects. Presently, our findings suggest that ADAM15 may play an important role in supporting the assessment of disease progression, particularly in cases with distant metastases. The importance of ADAM15 in distinguishing between subjects with the presence of distant metastases and patients without distant metastases was confirmed using nonparametric tests, as well as AUC analysis, which indicates its potential utility as a biomarker for metastatic spread. Moreover, we calculated the diagnostic criteria of serum ADAM15 in CRC patients to evaluate whether this protein might be used as non-invasive biomarker in the diagnostic process of CRC patients. Although the percentage of elevated ADAM15 concentration was lower than for classical tumor markers such as CEA and CRP, its diagnostic value notably increased when used in combination. Therefore, the combined analysis of ADAM15 with CEA or CRP may enhance the clinical evaluation of CRC patients, especially in the context of advanced disease or metastatic risk stratification.

This multi-marker approach could help to refine diagnostic sensitivity while capturing additional aspects of tumor biology not fully represented by classical markers alone. From a clinical perspective, the identification of biomarkers that predict metastatic potential is crucial for early risk stratification and individualized treatment planning in colorectal cancer. Although serum ADAM15 alone may not be sufficient for diagnosis, its significant elevation in patients with distant metastases highlights its potential role in metastatic risk assessment. In combination with markers such as CRP or CEA, ADAM15 may enhance diagnostic sensitivity and support clinical decisions regarding surveillance intensity and therapeutic strategies. These findings offer a rationale for the development of multi-marker panels and support future translational research into ADAM15 as both a biomarker and possible therapeutic target. Further research is warranted to confirm these observations and to define the most effective combinations for clinical application.

## 5. Limitations

This study has several limitations. The relatively small sample size may affect the statistical power and limit the generalizability of the results. The studied biomarkers tend to be labile, and measurements may be affected by degradation during processing, storage, and testing. However, our samples had been thawed only once before the current analysis. Serum samples from patients with chronic inflammatory conditions were not available in our study, which restricts the evaluation of marker specificity in clinically relevant differential diagnoses. However, we reported that some of the tumor patients (26%) who were negative for CRP presented positive concentrations of ADAM15. In addition, a positive correlation between soluble ADAM15 levels in serum and its expression in CRC tissue has been previously proven by other researchers. Thus, the aim of our study was to focus on the measurement of ADAM15 concentrations in the serum of CRC patients without parallel analysis in tumor tissue, as well as the mRNA or protein level. To evaluate whether soluble ADAM15 could potentially be used as a non-invasive biomarker to monitor cancer progression, especially distant metastasis, the ELISA method has been employed. ELISA is a widely used technique; however, it also has several limitations such as sensitivity issues, potential for background noise and cross-reactivity, and a limited dynamic range. Additionally, ELISA relies on antibodies, which can exhibit batch-to-batch variability and may not always be available for all target antigens.

## 6. Conclusions

There is a dearth of research on the serum concentrations of novel candidate biomarkers of CRC, because early diagnosis of this tumor through the assessment of serum biomarkers remains a great challenge for future medicine. Despite the knowledge concerning the upregulated expression levels of ADAM15 in CRC tissues found using the labor-intensive immunohistochemical method, there are no investigations assessing the serum concentrations of this protein in CRC patients. Thus, easy-to-perform, cost-effective, and non-invasive methods in the diagnosis of CRC are crucial for the further diagnosis of CRC patients. In conclusion, our results confirm the significance of ADAM15 in the pathogenesis of CRC, especially as a predictor of distant metastasis. Based on the diagnostic criteria, serum ADAM15 seems to be a candidate marker for CRC diagnosis, especially in combined use with a classic tumor marker—CEA. Further investigations are needed to clarify its potential use as a biomarker of CRC.

## Figures and Tables

**Table 1:** Characteristics of colorectal cancer patients.

Variable Tested		Number of Patients
Group	Colorectal cancer	110
	Control (healthy patients)	54
Gender	Male	82
	Female	28
TNM (tumor–nodulus–metastasis) stage	I	20
	II	36
	III	30
	IV	24
Depth of tumor invasion (T factor)	T1 + 2	26
	T3	67
	T4	17
Nodal involvement (N factor)	N0	62
	N1	46
	unknown	2
Distant metastases (M factor)	M0	86
	M1	24

**Table 2:** Serum levels of proteins tested in patients with colorectal cancer in comparison to healthy controls.

		ADAM15 (pg/mL)	CEA (ng/mL)	CA19.9 (U/mL)	CRP (mg/L)
Colorectal cancer patients	Mean	756.724	43.272	135.410	20.595
	Std. Deviation	491.555	204.536	878.717	40.128
	Minimum	33.769	1.730	2.060	1.000
	Median	774.760	2.455	4.460	6.100
	Maximum	2244.990	1500.000	9035.000	298.000
Healthy group	Mean	725.968	2.022	7.050	2.091
	Std. Deviation	394.160	1.664	7.938	2.653
	Minimum	50.925	0.500	2.000	0.200
	Median	686.493	1.730	4.090	1.200
	Maximum	1798.070	11.490	40.970	18.400
*p* (Mann–Whitney test)		0.885	<0.001	0.196	<0.001

Statistically significant when *p* < 0.05.

**Table 3:** Serum levels of biomarkers tested in colorectal cancer patients in relation to tumor stage.

TNM		ADAM15 (pg/mL)	CEA (ng/mL)	CA19.9 (U/mL)	CRP (mg/L)
I	Mean	532.864	5.408	6.926	14.125
	SV	419.384	8.339	8.930	31.658
	Minimum	82.235	1.730	2.060	1.000
	Median	335.056	1.730	4.000	2.250
	Maximum	1545.128	37.900	41.370	141.300
II	Mean	789.707	47.498	47.021	17.411
	S.D.	450.424	249.162	200.816	49.828
	Minimum	128.659	1.730	2.060	1.000
	Median	859.782	2.275	4.115	4.800
	Maximum	1947.119	1500.000	1200.000	298.000
III	Mean	695.508	66.052	80.716	15.577
	S.D.	473.989	273.573	277.064	20.864
	Minimum	117.303	1.730	2.060	1.000
	Median	708.289	2.675	5.635	7.450
	Maximum	1493.624	1500.000	1200.000	99.900
IV	Mean	970.319	40.013	443.433	37.038
	S.D.	556.798	86.042	1836.186	45.923
	Minimum	33.769	1.730	2.060	1.000
	Median	1005.632	6.845	5.480	13.700
	Maximum	2244.990	362.540	9035.000	149.000
*p* (Kruskal–Wallis)		0.081	0.049	0.387	0.013
*p* (test post hoc wg Dwass–Steele–Critch–ow–Fligner)	I vs. II		0.633		0.616
	I vs. III		0.388		0.349
	I vs. IV		0.051		0.024
	II vs. III		0.904		0.833
	II vs. IV		0.166		0.048
	III vs. IV		0.603		0.289

Statistically significant when *p* < 0.05.

**Table 4:** Serum levels of biomarkers tested in colorectal cancer patients in relation to clinicopathological features of tumor.

		ADAM15 (pg/mL)	CEA (ng/mL)	CA19.9 (U/mL)	CRP (mg/L)
T1 + 2	Mean	588.485	76.194	15.954	17.377
	Std. Deviation	423.360	298.826	37.474	38.727
	Minimum	82.235	1.730	2.060	1.000
	Median	418.729	1.730	5.600	3.200
	Maximum	1545.128	1500.000	194.310	149.000
T3	Mean	777.039	38.173	207.298	19.761
	Std. Deviation	491.589	185.787	1121.442	40.531
	Minimum	33.769	1.730	2.060	1.000
	Median	847.174	2.350	4.550	6.200
	Maximum	2244.990	1500.000	9035.000	298.000
T4	Mean	881.598	13.281	36.832	29.825
	Std. Deviation	507.496	16.310	124.999	43.093
	Minimum	150.498	1.730	2.060	1.000
	Median	1003.568	5.365	4.035	9.700
	Maximum	1853.037	56.190	505.280	141.300
*p* (Kruskal–Wallis test)		0.214	0.075	0.736	0.061
N0	Mean	722.540	29.748	39.417	15.263
	Std. Deviation	442.955	189.967	167.523	41.830
	Minimum	82.235	1.730	2.060	1.000
	Median	768.762	1.900	4.225	4.550
	Maximum	1947.119	1500.000	1200.000	298.000
N1	Mean	791.937	61.709	270.581	27.002
	Std. Deviation	558.891	227.598	1341.679	37.318
	Minimum	33.769	1.730	2.060	1.000
	Median	797.480	3.585	5.895	8.400
	Maximum	2244.990	1500.000	9035.000	149.000
*p* (Mann–Whitney test)		0.804	0.012	0.170	0.006
M0	Mean	697.116	44.182	49.451	16.007
	Std. Deviation	457.695	227.245	208.774	37.371
	Minimum	82.235	1.730	2.060	1.000
	Median	681.053	2.155	4.355	4.800
	Maximum	1947.119	1500.000	1200.000	298.000
M1	Mean	970.319	40.013	443.433	37.038
	Std. Deviation	556.798	86.042	1836.186	45.923
	Minimum	33.769	1.730	2.060	1.000
	Median	1005.632	6.845	5.480	13.700
	Maximum	2244.990	362.540	9035.000	149.000
*p* (Mann–Whitney test)		0.043	0.021	0.162	0.004

Statistically significant when *p* < 0.05.

**Table 5:** The statistical differences between the different stratified groups (TNM stage, T, N, and M factor) and the healthy controls.

		ADAM15 (pg/mL)	CEA (ng/mL)	CA19.9 (U/mL)	CRP (mg/L)
TNM stage I	*p*	0.016	0.039	0.975	0.022
TNM stage II	*p*	0.400	<0.001	0.656	<0.001
TNM stage III	*p*	0.650	<0.001	0.180	<0.001
TNM stage IV	*p*	0.044	<0.001	0.180	<0.001
T1 + 2	*p*	0.070	0.007	0.183	0.003
T3	*p*	0.584	<0.001	0.199	<0.001
T4	*p*	0.395	<0.001	0.583	<0.001
N0	*p*	0.894	<0.001	0.537	<0.001
N1	*p*	0.806	0.001	0.049	<0.001
M0	*p*	0.534	<0.001	0.403	<0.001
M1	*p*	0.043	<0.001	0.063	<0.001

Statistically significant when *p* < 0.05 in Mann–Whitney test.

**Table 6:** Diagnostic criteria for analyzed proteins.

	Sensitivity	Specificity	PPV	NPV	ACC
ADAM15	38	81	81	39	52
CEA	58	81	85	46	62
CA 19-9	52	96	92	37	45
CRP	65	91	93	56	73

**Table 7:** Logistic regression test results in relationship between risk factors and colorectal cancer (CRC) occurrence.

Univariate Logistic Regression Results				
	*p*	OR (odds ratio)	95% C.I. (confidence intervals)	
ADAM15	0.687	1.000	0.999	1.001
CEA	0.004	1.428	1.124	1.814
CA19.9	0.204	1.018	0.990	1.048
CRP	0.000	1.392	1.176	1.647
Multivariate logistic regression results				
	*p*	OR (odds ratio)	95% C.I. (confidence intervals)	
ADAM15	0.279	0.999	0.998	1.000
CEA	0.018	1.352	1.052	1.737
CA19.9	0.614	1.012	0.966	1.060
CRP	0.001	1.341	1.120	1.605

## Data Availability

Data available on request due to privacy/ethical restrictions.
